# A quantitative study of social identity, social support and perceived stress in online support groups for family caregivers

**DOI:** 10.1177/13591053251377890

**Published:** 2025-10-07

**Authors:** Rosemary Daynes-Kearney, Stephen Gallagher

**Affiliations:** 1 University of Limerick, Ireland

**Keywords:** family caregivers, perceived stress, online support groups, social support, social identity

## Abstract

Online support groups (OSGs) may help reduce family caregiver stress, but the psychosocial pathways remain unclear. Using social identity theory, this study examined the relationships between social identity, social support and perceived stress. It was hypothesised that non-OSG members would report lower social support and higher stress than OSG members, with social support mediating the relationship between social identity and stress. A cross-sectional online survey (*N* = 136) assessed social support, social identity and perceived stress. No significant differences in social support or stress were found between OSG (*n* = 78) and non-OSG members (*n* = 58), though OSG members identified more strongly as caregivers (*p* < 0.001). Higher social identity correlated with greater social support but not lower stress. Mediation analysis showed social support indirectly linked social identity to reduced stress. Findings highlight the role of social identity in OSGs and its potential for improving caregiver well-being.

## Introduction

The provision of care is one of the most important issues in society. Globally, 350 million people are care dependent (World Health Organization, 2017). Moreover, this figure continues to grow as people live longer (World Health Organization, 2024) and with better management of disabilities and chronic health conditions (Maresova et al., 2019). In most countries, family members provide care for children and adults with additional needs (World Health Organization, 2017), with the majority of caregivers being women (United Nations, 2022). Alongside this, there is a global deficit in the supply of professional caregivers, thus informal or family caregiving are now seen as a backbone of global healthcare systems (World Health Organization, 2017).

A family carer is a “person of all ages who provides care (usually unpaid) to family members, other relatives, partners, friends and neighbours with a long-term illness, disability or other long-lasting health or care need, outside a professional or formal employment framework” (Eurocarers, 2023). Across the board, the majority of family caregivers are women (Rocard and Llena-Nozal, 2022). In the EU, an estimated 10%–30% of people aged 18–75 are providing unpaid weekly long-term care (Zigante, 2018), while in the USA, this figure is nearly 16% of the adult population (National Alliance for Caregiving, 2020) and in Ireland, almost 300,000 people identified as family carers in the 2022 census (Central Statistics Office, 2023).

Rates of caregiving can also vary somewhat by ethnicity, and in the US, which is becoming more culturally diverse, 39% of adult caregivers identify as a racial or ethnic minority (Tran et al., 2023), with the highest group (21%) identifying as Hispanic compared to 17% identifying as White. Access to support can be more challenging for minority caregivers with evidence that they are less likely to utilise formal support services (Napoles et al., 2010) while the experience of LGBTI carers is not well understood (Eurocarers, 2025).

However, it is difficult to know how many family carers there are as many caregivers do not identify as such (Care Alliance Ireland, 2017), particularly when the care they are providing to is to a child or at the initial stages of the caregiving journey (Knepper and Arrington, 2016; Montgomery and Kosloski, 2013). Additionally caregiving motivations are deeply rooted in cultural values like filial piety, familism and religious beliefs (Zarzycki et al., 2022) and these different cultural experiences may impact the identification of family caregivers.

There are many positive achievements for family caregivers, such as the strengthening of existing relationships (Rapley et al., 2023), life learning and skill development (Tambunan and Simbolon, 2023) and resilience (Lök and Bademli, 2021). However, extensive research has demonstrated family caregivers often face health issues like back pain (Brennan et al., 2023), anxiety (Contreras et al., 2023) and depression (Gallagher and Wetherell, 2020; Piamjariyakul et al., 2024), higher blood pressure (Gallagher and Whiteley, 2012) and affected physiological biomarkers of stress (Zajdel et al., 2023). Carers often don’t prioritise their own health needs, with impacts on their own health and wellbeing. However, it can be argued that the critical source of the negative caregiver health impacts is the stress that family caregivers consistently report experiencing (e.g. Chesak et al., 2023, Daynes-Kearney and Gallagher, 2023, 2024, 2025; Pearlin et al., 1990).

Stress is a core topic within Health Psychology, with extensive research and practical initiatives dedicated to understanding and managing stress (Marks et al., 2021). Stress can be seen as a principal cause of psychological distress and physical illness in the general population. Research on stress and family caregivers specifically (e.g. Anderson et al., 1995; Hu et al., 2023; Moser and Dracup, 2004; Soylu et al., 2015), describe physical, emotional and psychological impacts on family caregivers. Psychosocial interventions have been shown to reduce many of the negative health effects of caring through improved coping skills and resilience or post-traumatic growth. Interventions using social support are a common tool to build resilience and support coping for family caregivers (Cantwell et al., 2015; Daynes-Kearney and Gallagher, 2025; Gallagher and Whitley, 2012; Naamara et al., 2022; Sabo and Chin, 2021).

Social support, another core concept rooted in Health Psychology, can be defined as “social interactions or relationships that provide individuals with actual assistance or that embed individuals within a social system believed to provide love, caring or a sense of attachment to a valued social group or dyad” (Hobfoll, 1988: 121). Social support has been shown to lead to positive impacts for family caregivers (Daynes-Kearney and Gallagher, 2023, 2024) and support groups are a tool for health promotion and ill-health prevention. The traditional model of support service provision for family caregivers has been in a face-to-face settings, however in person groups often present logistical challenges for family caregivers who, by nature of their role, are largely time restricted as well as often being unable to leave their loved one without respite care or a similar support service (Sharaievska and Burk, 2018). As a result, family caregivers have not always been able to avail of social support when they require it.

Online support groups (OSG) have been shown to be a viable place for family caregivers to source social support (e.g. Parker Oliver et al., 2014; Washington et al., 2020). With the development of telehealth and digital health services recent research has found that medical professionals often refer family caregivers to online support groups (Daynes-Kearney and Gallagher, 2023). OSG have the potential to draw together large numbers of people for the purposes of mutual peer support (Coulson, 2019) and are different from naturally occurring online exchanges as they are intentionally planned, have specific purposes, structures and rules or guiding principles (Washington et al., 2020). In practice, online support groups (OSG), have been utilised as support options both with formal state backing (e.g. Andréasson et al., 2018)or run by interest groups and non-governmental organisations or NGOs (e.g. *Care Alliance Ireland Online Support Group Ireland* and *The American Health Association in the USA*).

There is a wide body of research examining the positive effect of perceived social support in face-to-face settings such as in relieving caregiver burden (Wright et al., 2024), caregiver quality of life (Zhang et al., 2024) and positive coping for family caregivers (Yang et al., 2023) and in online settings such as helping to prepare for end -of-life care (Davies et al., 2020) and reducing social isolation (Daynes-Kearney and Gallagher, 2024). While there is a large corpus on OSG for patients (e.g. Rowlands et al., 2023; Setoyama et al., 2011; Smedley et al., 2015), the output for OSG for family caregivers is developing, and much is yet to be clarified (Daynes-Kearney and Gallagher, 2023), particularly in understanding why family caregivers may not join or remain in an OSG, a gap in knowledge this study seeks to address.

Additionally, while both the provision and receiving of social support has been demonstrated as a key resource for managing stress adaptions, there may be a positive, negative or indifferent reaction to stress (Hartley et al., 2022). This raises the question as to what way social support may influence stress for family caregivers. Relatedly, there is a gap in understanding the pathways of if and how OSG are an effective space in reducing the stress that caregivers experience. If OSG are to be used as a tool in health psychology interventions, it is vital to understand what makes them work and why. Therefore, this study sought to understand what psychosocial factors were important and how these factors related to perceived social support and stress.

In response to recent research which has highlighted the important of role of social identity for understanding the benefits derived from OSG (Daynes-Kearney and Gallagher, 2024), social identity was chosen as a theoretical framework to explore these questions under. Social identity is the knowledge and connection to groups that a person is a member of (Tajfel, 1978) and the Social Identity Approach (SIA) posits that group membership fosters social support, and those who have a stronger social identity with the group tend to derive the most health benefits (Haslam et al., 2009, 2018; Tajfel and Turner, 1981; Turner et al., 1987). Part of forming the identity of group membership comes when individuals identify with the norms, values or shared experiences of the group (Jetten et al., 2017). For members of OSG, the identification could help family caregivers move from seeing themselves as an isolated minority to recognising themselves as part of a collective.

The SIA has been used with different cohorts and has demonstrated a relationship between social support and social identity and health impacts. For example, McKimmie et al. (2020) illustrated that social support associated with high group identity had a positive mediating effect on stress. For people recovering from mental health illness, subjective identification with groups led to greater perceived social support and lower perceived stigma (Kearns et al., 2018). Additionally, a stronger sense of social identity and perceived social support is associated with reduced social isolation for stroke survivors (Lamont et al., 2023).

However, there has been inconsistent findings in the nature of the direction of relationship between social identity and social support. Most of the research including the SIA theory posit that social identity is what drives social support (Carter et al., 2023; Haslam et al., 2009), while others find that social support comes first (Akgül and Karafil, 2022; Zhang and Guo, 2023). Interestingly, researchers have also found reciprocal relationships with McKimmie et al. (2020) stating that social identity and social support were more concurrent perceptions rather than one leading to the other, and Ntonsis et al. (2023) finding that support mediated the relationship between social identification and stress and so there is a continued need to explore this relationship in this particular context.

Unambiguously for family caregivers, the role of shared experiences is important for the development of social support (Daynes-Kearney and Gallagher, 2024). However to our knowledge, there are limited studies in the caregivers OSG space which have examined whether members of OSG for family caregivers identify with their group members and whether this has an impact on the social support generated or perceived health benefits such as lowering stress. In applying the SIA to OSG for family caregivers, it could be posited that a higher social identity for family caregivers who are OSG members will be associated with reporting of higher social support and in turn lower perceived stress, a question this study seeks to explore.

As noted above, not all people who are engaged in caregiving activities may identify as a caregiver, and research has found that caregiver identity develops over time, often as caregiver burden increases (Montgomery and Kosloski, 2013). In considering previous literature, studies (e.g. Chien and Norman, 2009; Gräßel et al., 2010) have found that caregivers who attend social support groups tend to report higher levels of social support in comparison to those who do not attend, and consistent with SIA a stronger sense of identity with group membership confers higher social support, a higher sense of agency and reduced stress (Jetten et al., 2017). Consequently, it might be that family caregivers who are not members of OSG may also not identify as family caregivers. They may report less social support and higher stress compared to OSG members and these elements may be a result of them not being members of an OSG and not benefiting from this support.

In summary, OSGs could be a valuable tool for health psychologists and practitioners to support family caregivers with their levels of stress. However, there are gaps in this knowledge base that need to be explored to determine if they are a viable and worthwhile in this area. Therefore, the purpose of this research was to explore whether online support groups generate health benefits for users using perceived stress as a measure of health. A second area of exploration was to examine the relationship between social identity with OSG membership for family caregivers, social support and perceived stress to understand how OSG may reduce perceived stress.

Drawing the factors of identity, social support and their potential relationship to stress together, we hypothesised first that family caregivers who were not in an OSG would report lower levels of social support in general, be less likely to identify as a family caregiver and report higher stress compared to family caregivers in an OSG. Second, that for family caregivers in OSGs there would be a positive relationship between social identity with the OSG members and social support and that these would be associated with reporting of lower stress in this group. Third, and consistent with SIA theory, we anticipated that the association between OSG social identity and stress in family caregivers would be mediated by social support. However, given that others have found social support is what drives social identity we also examined this alternative path, that is that the association between social support and stress would be mediated by social identity. We also aimed to add knowledge about a hard-to-reach group, family caregivers who are not members of OSG to understand more about their social support and health as well as why they were not members of groups and whether identification as a family caregiver as a social identity had a role in this.

## Method

### Participant recruitment, study design and procedure

The study was a cross-sectional online survey of family carers (*n* = 136). This study received ethical approval [EHS 2021_11_07] and was registered on Open Science Framework (OSF) (https://osf.io/rvbx3/). All data participants were provided with information before taking the survey, including summary information and a link to more detailed information. Data participants gave written informed consent prior to participation by actively and voluntarily ticking a box to confirm that they understood the information and were happy to participate in the study. Data participants could close the survey at any time if they didn’t wish to continue with the survey.

The survey (see Supplemental File A) was developed with input from family caregiver groups (e.g. piloted tested the survey for timing and clarity), hosted using Qualtrics and was distributed via Care Alliance Ireland, Family Carers Ireland and other groups on their platforms and social media channels (e.g. Twitter and Facebook). The authors had previously completed qualitative research with members of an Irish online support group (Daynes-Kearney and Gallagher, 2023, 2025) and there was a fear of the same people completing the survey because of the small population in Ireland with an even smaller cohort of family caregivers. As such, the decision was made to open the study to family caregiver groups outside of Ireland. Nationality was gathered using a free-text box, where users could type their nationality.

The majority of respondents were Irish (*n* = 112), with other respondents identifying as American (3), British/English/UK/Ango-Irish (13), Australian (1), French (1), Greek (1), Indian (1), Polish, (1), Portuguese (1), Scottish (1) and Spanish (1). 120 respondents lived in Ireland and 16 lived in other countries, most in the UK (8). Users could choose their ethnic identity from a list which was developed from the Irish Central Statistics Office, with an option to type in their ethnic identity if not included in the list or not to include this information if they wished. Users could choose more than one ethnic identity. The majority of respondents identified as White (105).

### Measures

Socio-demographics and care-related variables (e.g. how long caring, type of illness person has, are you a member of an OSG, if not, why not etc), were measured by questions asking respondents to choose from a list of drop-down options. The questions and options were developed in line with literature that details important contextual factors for family caregivers and in consultation with family caregiver groups. Construct validity was tested on caregiver social identity, hours caring, employment, number of conditions, age, gender, nationality, how many years caring and whether the respondent was living in Ireland.

An exploratory factor analysis (EFA) was conducted using principal axis factoring with oblimin rotation. The Kaiser-Meyer-Olkin (KMO) measure of sampling adequacy was 0.594, indicating marginal suitability for factor analysis, while Bartlett’s test of sphericity was significant (χ^2^(28) = 72.77, *p* < 0.001). The initial analysis extracted three factors with eigenvalues greater than one, explaining 39.5% of the total variance. A review of the matrix showed that most variables loaded meaningfully onto the first two factors. Factor 1 included nationality and living in Ireland, reflecting a socio-demographic context factor. Factor 2 included social identity, age and caregiving experiences variables, consistent with caregiving context factors. The third factor was weakly defined, and the extraction required more than 25 iterations, indicating instability. Gender did not meaningfully load on any of the three extracted factors. These findings offer preliminary support for the construct validity of the in-house survey. However, due to issues with instability and interpretability, a revised two-factor model was tested.

The two-factor model demonstrated improved interpretability and clearer item loadings and converged after 21 iterations. The initial analysis again extracted three factors with eigenvalues greater than one, this time explaining 57.5% of the total variance In this solution, nationality and living in Ireland loaded strongly onto Factor 1 and Factor 2 included meaningful loadings from caregiver identification, age, years caring. The two-factor model showed a more stable solution with cleaner item loadings and clearer conceptual boundaries, providing stronger support for construct validity.

#### Social support

The Medical Outcomes Social Support (MOSS) (Sherbourne and Stewart, 1991) was used as a measure of social support. The standard MOSS is a 20-item self-reported measure of social support with four sub-scales measuring emotional/informational support, tangible support, affective support and positive interaction. Participants are asked to rate how often they perceived the availability of support to them over the previous month. The scale is scored from 1 (none of the time) to 5 (all of the time), the highest score possible was 55 with a low score considered below 20. The MOSS has been widely validated across diverse populations, including caregivers, supporting its construct validity. In this study, we used two subscales, emotional/informational support and positive interaction subscale as these have been identified as the most common types of support reported by family caregivers (Daynes-Kearney and Gallagher, 2025) and are key predictors of health outcomes in family caregivers (Puga et al., 2023) with Cronbach’s α = 0.95 for this study.

#### Perceived stress

The 4-item perceived stress (PSS-4) (Cohen et al., 1983) was used as a measure of stress in family caregivers. The scale asks participants to indicate how often they felt a factor over the previous month using responses ratings from 0 (never) to 4 (very often), with questions 2 and 3 reverse coded. A score over 12 indicates high perceived stress. The scale has been used previously in research with family caregivers, demonstrating good construct validity (e.g. Kunicki et al., 2021, Leong et al., 2024) and it has an excellent reliability, with Cronbach’s α = 0.86 in the present study in line with established benchmarks.

#### Caregiver identity and social identity

To explore social identity, we used a 6-item Social Identity Measure based Mael and Ashforth’s (1992) organisational identification scale which has been used to test social identity in online support groups (e.g. Dailey and Zhu, 2017; Zhu and Stephens, 2019). This measure asks questions such as “When I talk about this group, I usually say ‘we’ rather than ‘they’,” with responses rating from 0 (strongly disagree) to 5 (strongly agree). These items had *M* = 5.85, SD = 1.14, and a Cronbach’s α = 0.95. While adapted from an organisational context, the Social Identity Measure has been previously validated for use in online support groups (Dailey and Zhu, 2017), supporting its construct validity in this setting.

Two separate question was provided with a definition of a family carer, where respondents could choose first if they were a family caregiver (yes/no/don’t know) and second how strongly they identified as a family caregiver, ranging from not at all to very much.

#### Data analysis

All data was transferred to SPSS and data was recoded as directed in the standardised tools. Additionally recoding of text items to numeric items was completed. All recoding was completed by the first author in consultation with the second author. Prior to analysis data was screened and checked for outlier and normality. No outliers were identified and assumptions of normality met. Initially, frequency counts were used for demographic information. Analysis then moved to tests of difference and Pearson’s correlation to explore relationships between different variables. Further analysis was completed using linear regression analysis (controlling for co-variates) and mediation analysis (Process Model 4) to test whether social identity was a mediator in the relationships between social support and perceived stress. The data set will be made available on OSF when further analysis has been completed.

## Results

### Descriptive statistics

The survey had 206 respondents with 136 of those fully completing the survey. In terms of gender, 93.38% of the respondents identified as female, 6.61% as male and one chose not to share their gender identity (see Table 1 for characteristics). The age ranged between 19 and 72, mean age was 47 years, with 41.2% aged between 41 and 50 and 34.6% aged between 51 and 65. There was a wide range of conditions requiring care, with 69.9% of respondents indicating multiple conditions for their cared for person(s). 74 respondents (54.4%) identified a parental relationship to a person they cared for, with the next highest role (40%) that of a son or daughter/in law (*n* = 57). Of the 136 respondents, 78 reported being a member of an OSG and 58 reported no membership of an OSG. The highest given reason for non-membership was that they didn’t know about OSG (20.7%) while 15.5% didn’t have time or had other priorities.

*Q1*: Do members of OSG and non-OSG members differ on social support, perceived stress and caregiver identity?

**Table 1.** Demographics of survey participants.

Demographic item	Number of participants (*N* = 136)
Gender	(*N* = 136)
Female	127
Male	9
Prefer not to say	1
Age range (*M* = 47)	(*N* = 136)
19–24	10
24–40	17
41–50	56
51–65	47
Caring relationship	(*N* = 136)
Parent or parent-in-law	74
Child	57
Spouse or partner	11
Geographic area	(*N* = 136)
Living in Ireland	121
Other	15
Race/Ethnic identity	(*N* = 136)
White	106
Other	30
Irish	112
Other	24
Length of time caring	(*N* = 136)
15 Years or less	44
16 Years or longer	92
Hours of care per week	(*N* = 136)
Less than 20 hours	20
21–40 Hours	15
40+ Hours	101
Work and study	(*N* = 136)
Working and caring	45
Studying and caring	17
Gave up work/study due to caring role	39
Other	35
Online Support Group (OSG) membership	*N* = 78
Engaged daily	39
Members of more than one group	66
Demographic item	Number of participants (*N* = 136)
Members of a Facebook Group	48
Reasons for non-membership in OSG	*N* = 58 (more than one could be chosen)
Unaware of groups	12
Lack of time	9
Other priorities	9
Sufficient family/friend support	5
Other	23

In exploring the relationship between social support, social identity and stress in online support groups (OSG) for members and non-members of OSG, we first needed to understand whether there were differences between respondents who were members of online support groups (*n* = 78) and those that were not (*n* = 58) on socio-demographics, caring and variables of interest (see Table 2).

**Table 2.** Variables between non-OSG and OSG members.

Variable	Non-OSG members (*N* = 58)	OSG members (*N* = 78)	Test of difference
Age (51–65), %	22.5	42.7	χ^2^(4) = 22.90, *p* < 0.001**
Gender (female), %	93.8	92.6	χ^2^(1) = 0.08, *p* = 0.77
Nationality (Irish), %	88.9	79.3	χ^2^(1) = 2.14, *p* = 0.14
Ethnicity (White), %	77.7	75.6	χ^2^(1) = 0.085, *p* = 0.77
Caring for one person, %	74.1	56.1	χ^2^(2) = 5.00, *p* = 0.08
Care recipient (parent), %	44.4	40.2	χ^2^(8) = 24.32, *p* = 0.145
Years caring (16+), %	18.9	41.5	χ^2^(1) = 7.48, *p* = 0.006*
Hours caring per week (21+), %	9.8	22.2	χ^2^(1) = 4.034, *p* = 0.045*
Number of conditions (2+), %	61.1	76.6	χ^2^(1) = 3.25, *p* = 0.071
Working and caring (yes)	48.1	32.9	χ^2^(1) = 3.172, *p* = 0.075
Idenfication as carer	3.31 (0.91)	3.78 (0.55)	*t*(1134) = 3.73, *p* < 0.001**
Social support generally	33.11 (11.46)	35.70 (12.50)	*t*(1134) = 2.21 *p* = 0.11
Perceived stress	12.87 (1.80)	12.91 (1.40)	*t*(1134) = 2.21 *p* = 0.16

Significant effects are highlighted in bold.

* *p* < 0.05 level. **p < 0.01 level.

As can be seen in Table 2, that there is a significant difference in age between OSG members and non-members, with a higher percentage of 51- to 65-year-olds being an OSG member. In addition, a higher proportion of OSG members reported caring for 16 years or more and caring for 21 hours or more per week compared non-group members. There was no difference in how many people the respondent was caring for, the relationship between the respondent and the cared for person or whether the respondents were working or not. Although not significant, it is interesting to note that a higher percentage of non-OSG members were working and caring for one person, which may indicate a lower level of caregiver burden but more pressure coming from occupying multiple life roles.

Of our main variables of interest, there was no significant group differences for general social support nor on perceived stress. Both groups had mean scores over 12 which indicates that members and non-members had high levels of perceived stress even with good levels of social support (both social support group means were over 30). This indicates that membership or non-membership of OSG may not have an impact on perceived stress and that social support may also be found in other structures outside online support groups.

However, OSG members did identify more strongly as family carers. Given the group differences on age, hours and years caring, we checked to see if our group differences on caregiver identity were still evident after controlling for these. Further, we also checked to see if these potential confounding factors were masking any group differences on general social support and perceived stress.

Using hierarchical linear regression analysis, the confounding factors above were entered in Step 1, and OSG membership was added at Step 2, followed by each of the variables of interest as the dependent variable, that is, carer identity, general social support and perceived stress. For carer social identity, we found that identification of a family caregiver became non-significant in Step 2, with β = −0.17, 95% CI [−0.519, 0.002], *t* = −1.97, *p* = 0.052. In this model, both hours β = −0.24, 95% CI [−0.829, −0.175], *t* = −3.03, *p* = 0.003, and years caring β = 0.18, 95% CI [0.012, 0.555], *t* = 2.06, *p* = 0.041, were significant predictors of carer identity such that the higher hours caring was negatively associated and weakened their caregiver identity, while those caring for longer (16 years or more) had a stronger identity. In the analysis for general social support, controlling for these potential confounds made no difference to the non-significance found for these variables in Tabel 17, both *p*’s > 0.10.

In summary, members of OSG tended to be older, caring for longer and caring for more hours across a week. They were more likely to be caring for more than two people but less likely to be working and caring. There was no differences between levels of perceived stress or social support for OSG and non-OSG members, with both groups having a high level of perceived stress and medium level of social support. However, OSG members were more likely to identify as a family caregiver than non-OSG member.

*Q2*: Within OSG analysis: Are there associations between OSG social support, OSG social identity and stress in family caregivers

We next considered the relationships between our main variables of interest and key demographic factors of members of online support groups. However, to check for potential co-variates we ran a correlation analysis (see Table 3) and there were several positive associations between age and nationality, age and years caring, nationality and hours caring per week. Irish carers were more likely to be older and caring for longer, however, for nationality, the respondents were overwhelmingly Irish and this therefore could skew the prevalence of these positive correlations. Older carers were more likely to be caring for longer but engaged less frequently while working was negatively correlated with number of hours caring. Those who were caring for more conditions were also more likely to be working.

**Table 3.** Correlations across socio-demographic, social support, stress, social identity and participation factors for OSG members.

Variable	1	2	3	4	5	6	7	8	9	10	11
1. Age	–										
2. Gender	0.02	–									
3. Nationality	**0.178** *	−0.145	–								
4. Years caring	**0.471** **	0.088	0.030	–							
5. Hours caring p/w	−0.126	−0.11	**0.256** **	−0.058	–						
6. No of conditions	0.022	0.018	−0.088	0.117	0.156	–					
7 .Working (yes or no)	−0.007	−0.038	−0.094	−0.008	−0.194*	0.156	–				
8. Frequency engagement	−0.232*	−0.014	0.127	0.062	0.045	−0.029	−0.023	–			
9. Total social support	0.047	−0.153	0.055	−0.080	0.099	−0.014	0.099	−0.145	–		
10. Social identity with OSG	0.067	0.044	0.044	−0.133	0.158	−0.026	0.158	−0.164	**0.64** **	–	
11. Perceived stress	−0.136	0.090	−0.035	−0.006	0.145	−0.056	0.145	0.214	0.014	0.13	–

Significant effects are highlighted in bold. Statistics refer to Pearson’s Moment Correlations.

* *p* < 0.05 level. **p < 0.01 level.

Considering the main variables of interest, there was a significant positive relationship between social identity and support but not between social identity and stress, or social support and stress (see Table 3). This indicates that a stronger social identity with the group was associated with higher social support, but that neither social identity nor social support was associated with perceived stress.

**Figure 1.**
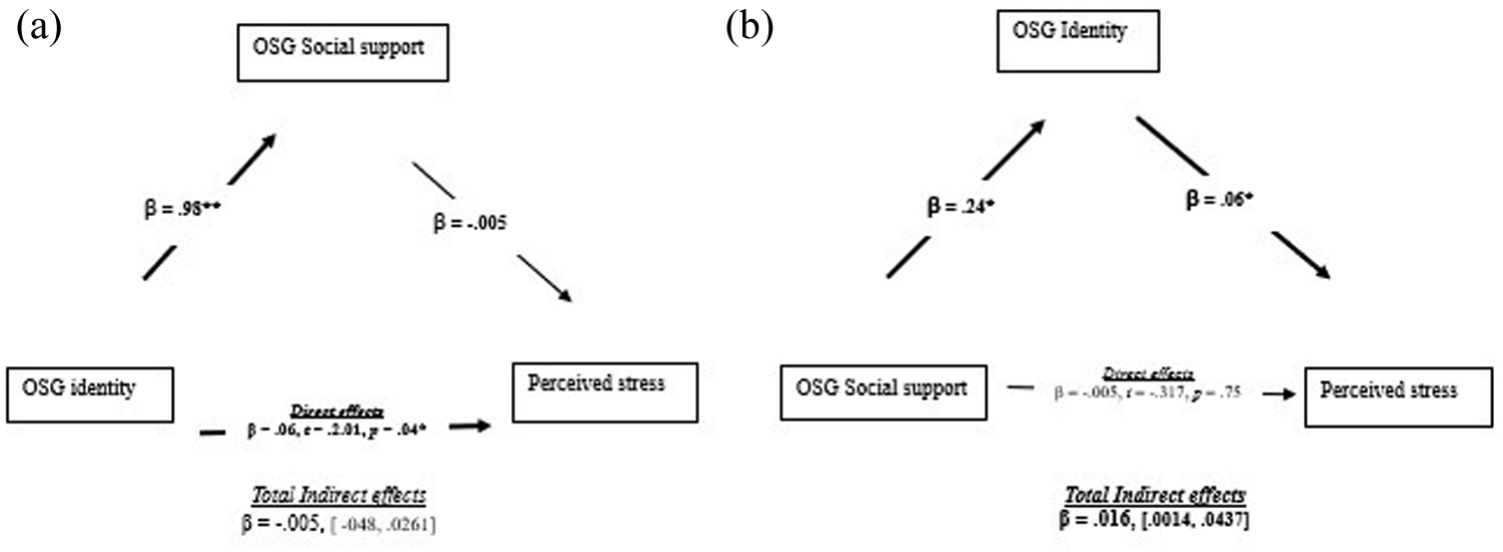
Mediation analysis (*N* = 78) of the relationship between (a) OSG member identity, OSG social support and perceived stress and (b) OSG social support, OSG member identity and perceived stress Significant effects are highlighted in bold. Statistics refer to standardized betas (β) and 95% confidence intervals at the lower and upper limit for total indirect effects. *p < 0.05 level. **p < 0.01 level.

For the mediation analysis, we controlled for the co-variates above. For the first model, OSG social identity was the predictor, social support from OSG members was the mediator and perceived stress was the outcome (see Figure 1(a)). As can be seen, there was a positive association between social identity and social support such that a higher social identity with OSG members was associated with a higher reporting of social support from OSG members. However, social support from members was not correlated with perceived stress. Further, there was a direct, albeit small effect and positive relationship between OSG social identity and perceived stress, such that those with a higher identity with their OSG members reported lower social support. We also tested the alternative model (Figure 1(b)), whereby OSG social support was the predictor, OSG social identity was the mediator and stress was the outcome. As can be seen, in this model the mediation model was significant, such that those reporting higher social support from OSG members, also reported a higher social identity, and in turn this was associated with lower stress.

## Discussion

Stress and its impacts and interventions to mitigate the effects of stress are a core topic in Health Psychology. Family caregivers, as a cohort, are evidenced to experience high levels of stress and online support groups (OSG) are one method that can be used to provide social support to them, as social support has been shown to have a positive effect in coping with stress. The main purpose of this study was to explore whether OSG would have an impact on the perceived stress experienced by family caregivers and to explore the relationship between social support and social identity and perceived stress. A second aim was to see whether family caregivers who were not in OSG would differ from those who are in OSG, on levels of social support perceived stress. However, in relation to the latter we did not find this as there was no difference between the groups.

For our within OSG group analysis we did find that social support and social identity were positively correlated such that those with higher OSG social support reported higher social identity with OSG members. On its own, social support from OSG members was not directly correlated with perceived stress, but social identity was, such that those reporting a higher social identity with OSG members also reported lower perceived stress. Moreover, we did not find evidence for mediation from social identity through social support to stress. Interestingly the alternative mediational model was significant such that those reporting higher social support from OSG members, also reported a higher social identity and in turn this was associated with lower reporting of perceived stress.

These results present a novel finding for a hard-to-reach group of family caregivers that is those who don’t participate in OSG, and the level of social support and perceived stress they are experiencing. While several other studies have found that family caregivers who attend social support groups tend to report increased levels of social support and lower stress (Chien et al., 2011; Gräßel et al., 2010), this was not the case here. Unexpectedly, we found that those attending OSG did not differ on general social support or perceived stress when compared to those who were not members. While we found no significant correlation between role and the factors of interest, there was a high percentage of respondents who were parents and perhaps they were getting support from parental groups. It would be interesting to further explore the alternative sources of support for family caregivers by their relationship to understand this further.

Considering why there may be no differences between the groups, the average score per group was in the mid-range of social support which indicates that non-OSG members may be getting support from other sources than OSG. Of this group however, there were few who said they had enough support from family and friends. This indicates that more work on where social support is generated is warranted and how it may change in different contexts. In fact, a review of the literature on social support groups for family caregivers noted that the majority in this area are cross-sectional in nature with few studies of a longitudinal nature and as such it is hard to disentangle the effects to see if those who join are low in social support initially or maybe those who do not join have relatively higher levels of social support already (Chien and Norman, 2009).

Both OSG members and non-members reported high levels of stress. Here, there were some key demographic differences between OSG members and non-members, with OSG members tending to be older, caring for longer and for more hours weekly. This aligns with research showing OSG have been found to be most useful for caregivers who are time-limited and unable to freely leave their cared-for person (Daynes-Kearney and Gallagher, 2023, 2024, 2025). Non-OSG members were more likely to be working and caring and caring for more than one person and may fall more into the category of “sandwich caregivers” (Miller, 1981). Sandwich caregivers face demands from caring for children, parents and employment, and suffer a paucity of time to take care of themselves (Steiner and Fletcher, 2017) with women most at risk in these roles.

These observations match with those of this current study where many of the non-OSG respondents felt they had other priorities or didn’t have time to engage in OSG. Therefore, while the perceived stress scores in this study may be similar, it is possible that the sources of stress may be different for each cohort, with OSG members experiencing stress from high care workload, poor opportunities for social engagement and social isolation while non-OSG members may be under the strain of myriad competing demands.

Of note, becoming a family caregiver can also have a significant impact on a person’s identity, as they are now often focused on the cared-for person with a loss of sense of self (Kruk, 2015). Our results found that OSG members identified more strongly as a family caregiver, which may make them more likely to seek support (Care Alliance Ireland, 2017; Dobrof and Ebenstein, 2004). Based on previous literature, caregiver identity may get stronger the longer one is caregiving or with higher levels of caregiver burden (Montgomery and Kosloski, 2013). This stronger identification may also affect the quality of support received as the social identity approach posits that the weaker self-identification of non-group members affected the quality and quantity of support used in other settings (Hartley et al., 2022) and in online community for family caregivers (Prato et al., 2022).

In terms of SIA theorical framework for understanding the psychosocial pathways behind the benefits of OSGs, we explored the relationship between OSG social support and OSG social identity and stress. Our non-significant result for a direct effect of social support on perceived stress is not in line with other studies (e.g. Lovell and Wetherell, 2023) although it is worth noting that other caregiver studies have argued that perceived support is less likely to influence caregiver health, rather they often need more formal support such as respite to cope with some of their caregiver challenges (Cantwell et al., 2014).

However, we did find a direct association between OSG social identity and perceived stress in OSG members, such that those reporting a higher level of identification with their OSG members also reporting a lower level of stress. This finding is similar to that found elsewhere for face-to-face support groups (Wakefield et al., 2013) and those living in rural areas (Carroll et al., 2019) where those who identified more strongly with their group members reported lower levels of distress. Consistent with SIA prediction (Haslam et al., 2009, 2018) and with other literature (e.g. Carroll et al., 2019), we found a positive correlation between OSG social support and OSG social identity.

Our key aim was to apply the social identity approach to better understand the psychosocial pathways behind OSG health benefits. Our hypothesis, driven by a SIA frameworks was that social support from OSG members would mediate the association between OSG social identity and stress but we failed to find support for this particular model. Instead, the alternative model was evident whereby social identity was the mediator between OSG social support and perceived stress. Those family caregivers who were OSG members and reported higher levels of support from this group, also had a stronger social identity with this OSG group reported lower levels of stress. Even though we have evidence for the alternative model, other SIA studies have also found similar evidence for this model (Akgül and Karafil, 2022; Zhang and Guo, 2023) and given the cross-sectional nature of the study, it does suggest that more research is required in this area.

The SIA approach has come to play a prominent role in health psychology through the theory of the Social Cure (Haslam et al., 2018; Wakefield et al., 2022) and the use of social prescribing, which focus on the health benefits of being a member of many groups with many health services using this as a tool to combat social isolation and loneliness and help maintain health (e.g. see the Irish Health Service Executive (2022) Social Prescribing Framework). Our results support existing work in this area, where Zhu and Stephens (2019) found that social identity was a stronger mediating factor for participation and social support than other types of group bonds, while online group identity should be cultivated through active moderation by trained peer or professional moderators (Daynes-Kearney and Gallagher, 2024; Prato et al., 2018).

Despite the novelty of the study, there are several limitations to this study worth mentioning. Firstly, as can be seen in Table 1, the respondents were predominantly White, Irish and female. While online supports are often positioned as a method of support hard to reach populations and we used known gatekeeper organisations and social media outlets to try and have a wide spread of reach, our study does not seem to have attracted responses from minority groups. Family caregivers are diverse, with race and gender affecting caregiving impacts (Cohen et al., 2019) and access to and use of supports. Unfortunately, while we can highlight this issue, our study does not provide any more information on how OSG may be or may not be used by family caregivers from minority backgrounds.

There was an incompletion rate of approximately 35% of the survey. While this is within an acceptable range, it does require reflection on the survey design and whether there were barriers to completion that were not considered especially the length of the survey. This is a point that will be taken forward into future design. Additionally, in line with the feedback from the pilot, the demographic information was gathered at the end. This means that analyses of the characteristics of who dropped off could not be completed, which could have provided additional feedback on who was interested in the survey and reveal whether more minority caregivers started but didn’t complete the survey. Given the cross-sectional nature of the study we cannot infer causation. This was a quantitative study and it would be worthwhile for qualitative studies in this area to be completed. Finally, as can be seen from OSG membership engagement reporting, there are several reasons why people engage with OSG. As the processes underling identity with OSG are to be fully elucidated, as such longitudinal studies may be important for this element.

## Conclusion

This study found that member of OSGs do not differ on levels of social support or perceived stress relative to non-OSG member. However, members of OSG did identify more strongly as family caregivers and perhaps this offers a reason why they may join OSGs. Importantly, we have shed light on some of the underlying processes linking OSG to health in that we have found that higher levels of social support from OSG members was associated with higher social identity which in turn predicted lower levels of perceived stress. These results have practical applications for those who set-up and run OSG for family caregivers as they demonstrate that identity with the OSG is a central component in the generation of social support and that work needs to be undertaken to actively develop a person’s sense of identity with the group. They also show the value of OSG as a tool for positive health benefits for family caregivers, which should be considered in any formal policy or strategy in digital health initiatives for family caregivers.

## Supplemental Material

sj-pdf-1-hpq-10.1177_13591053251377890 – Supplemental material for A quantitative study of social identity, social support and perceived stress in online support groups for family caregivers Supplemental material, sj-pdf-1-hpq-10.1177_13591053251377890 for A quantitative study of social identity, social support and perceived stress in online support groups for family caregivers by Rosemary Daynes-Kearney and Stephen Gallagher in Journal of Health Psychology
