# Supplementary material for: A quantitative study of social identity, social support and perceived stress in online support groups for family caregivers
Source: J Health Psychol. 2025 Oct 7;31(5):2080–97. doi: 10.1177/13591053251377890 (PMC13031349; doi:10.1177/13591053251377890)
Supplement: sj-pdf-1-hpq-10.1177_13591053251377890 – Supplemental material for A quantitative study of social identity, social support and perceived stress in online support groups for family caregivers [file sj-pdf-1-hpq-10.1177_13591053251377890.pdf]

Informed_Consent

Thank you for your interest in this survey.

We are carrying out a study on the experiences of Family Carers/Caregivers using online social support groups.

This is an anonymous online survey about your experience of using online support groups or if you are not a member of an online support group, why you are not.

It will help us understand how online support groups operate, what works well and any challenges for family carers/caregivers when using online social support groups.

It should take approximately 15 minutes to complete this survey.

To participate in this survey, please click the appropriate box below. You may withdraw your consent to participate at any time, without obligation by simply closing the survey. If you do this, no answers you have provided will be stored.

If you do not wish to participate in this survey, click the appropriate box below and you will exit the survey.

Please note, you must be over 18 to take part in this survey.

More information about this study can be found by copying and pasting this link https://bit.ly/surveyinfomation

Research Privacy Notice can be read by copying and pasting this link https://bit.ly/surveyprivacynotice

- ○ I confirm I am over 18 and I understand what is outlined above and I consent to opt-in to the survey
- ○ No thanks, I do not wish to participate

If you would like to be contacted in the future about a follow-up to this survey, please include your email here.

Block 10

Thank you for your interest in this survey.

As you have not given consent, the survey will end here

Carer ID

A family carer or family caregiver is anyone, including children and adults who looks after a family member, partner or friend who needs help because of their illness, frailty, disability, a mental health problem or an addiction and cannot cope without their support.

For the purposes of this survey, we will use the term family caregiver.

I am a family caregiver.

- Yes
- No
- Don't know

How strongly do you identify with being a family caregiver?

- Not at all
- Somewhat
- Quite a bit

○ Very much

Donot_ID_Caregiver

Thank you for your interest in this survey.
As you are not a family caregiver, the survey will end here.

Mastery

In this first set of questions, think about whether you agree or disagree with the given statement.
Choose the option that is most characteristic of you usually in your life in general.

There's no way that I can solve some of the problems that I have.

○ Strongly disagree
○ Disagree
○ Somewhat disagree

- ◯ Neither agree nor disagree
- ◯ Somewhat agree
- ◯ Agree
- ◯ Strongly agree

Sometimes I feel that I am being pushed here and there in life.

- ◯ Strongly disagree
- ◯ Disagree
- ◯ Somewhat disagree
- ◯ Neither agree nor disagree
- ◯ Somewhat agree
- ◯ Agree
- ◯ Strongly agree

I have little control over things that happen to me.

- ◯ Strongly disagree
- ◯ Disagree
- ◯ Somewhat disagree
- ◯ Neither agree nor disagree
- ◯ Somewhat agree
- ◯ Agree
- ◯ Strongly agree

I can do anything when I put my mind to it.

- Strongly disagree
- Disagree
- Somewhat disagree
- Neither agree nor disagree
- Somewhat agree
- Agree
- Strongly agree

Most of the time, I feel helpless when dealing with problems of life.

- Strongly disagree
- Disagree
- Somewhat disagree
- Neither agree nor disagree
- Somewhat agree
- Agree
- Strongly agree

What happens to me in the future mostly depends on me.

- Strongly disagree
- Disagree

- ○ Somewhat disagree
- ○ Neither agree nor disagree
- ○ Somewhat agree
- ○ Agree
- ○ Strongly agree

There's little I can do to change most of the important things in my life.

- ○ Strongly disagree
- ○ Disagree
- ○ Somewhat disagree
- ○ Neither agree nor disagree
- ○ Somewhat agree
- ○ Agree
- ○ Strongly agree

Social_Support_General

People sometimes look to others for companionship, assistance, or other types of support.

How often is each of the following kinds of support available to you in general?

	None of the time	A little of the time	Some of the time	Most of the time	All of the time
Someone you can count on to listen to you when you need to talk	○	○	○	○	○
Someone to give you information to help you understand a situation	○	○	○	○	○
Someone to give you good advice about a crisis	○	○	○	○	○
Someone to confide in or talk to about yourself or your problems	○	○	○	○	○
Someone whose advice you really want	○	○	○	○	○
Someone to share your most private worries and fears with	○	○	○	○	○
Someone to turn to for suggestions about how to deal with a personal problem	○	○	○	○	○
Someone who understands your problems	○	○	○	○	○
Someone to have a good time with	○	○	○	○	○
Someone to get together with for relaxation	○	○	○	○	○

	None of the time	A little of the time	Some of the time	Most of the time	All of the time
Someone to do something enjoyable with	○	○	○	○	○
Someone to do things with to help you get your mind off things	○	○	○	○	○

QOL_Question

How satisfied are you with life these days?

Click the number that best applies.

1 means very dissatisfied, 10 means very satisfied.

- ○ 1
- ○ 2
- ○ 3
- ○ 4
- ○ 5
- ○ 6
- ○ 7
- ○ 8
- ○ 9
- ○ 10

Membership of Support Groups

Thinking about your social engagement, are you a member of any caregiver support groups, either online or face-to-face?

- ○ Yes
- ○ No

Are you a member of any online support groups?

- ○ Yes, I am a member of online and face to face support groups
- ○ Yes, I am a member of online support groups only
- ○ No, I am not a member of any online support groups

How many online support groups are you a member of?

- ○ 1
- ○ 2
- ○ 3
- ○ 4
- ○ 5
- ○ 6

- ○ 7
- ○ 8
- ○ 9
- ○ 10+

How many online support groups that you are member of are dedicated to supporting family caregivers?

- ○ None
- ○ 1
- ○ 2
- ○ 3
- ○ 4
- ○ 5
- ○ 6
- ○ 7
- ○ 8
- ○ 9
- ○ 10+

Where do you engage with these family caregiver support groups?
Click all that apply

- ☐ Facebook public group
- ☐ Facebook private group

- ☐ Twitter
- ☐ Instagram
- ☐ Other Social Media platform
- ☐ Online general public forum e.g. boards.ie
- ☐ Online specific forum e.g.
- ☐ Forum linked to support service website
- ☐ Zoom meetings
- ☐ ____________ Other

How did you find out about the groups?
Click all that apply.

- ☐ Came up in my timeline
- ☐ Saw it advertised
- ☐ Recommended to me by family/friends
- ☐ Recommended to me by a service provider
- ☐ ____________ Other

Think of one online support group for family caregivers that you are a member of.

This can be a group that you engage in frequently or infrequently.

You can have had positive and/or not so positive experiences of this group

In what way do you engage with the group?

Click all that apply.

- ☐ I read content posted by others
- ☐ I post my own questions in the group
- ☐ I answer questions in the group
- ☐ I welcome new people in the group
- ☐ I express my empathy to people's posts in the group
- ☐ I give suggestions to people in the group
- ☐ I look for new friends in the group
- ☐ I seek new information in the group
- ☐ I connect people with others in the group
- ☐ I send private messages to other members of the group (not moderators)
- ☐ I send private messages to moderators in the group
- ☐ I follow discussion threads in the group
- ☐ I attend virtual events via videoconferencing e.g. Zoom
- ☐ I don't engage with the group
- ☐ __________ Other

How often do you engage with this group?

- ○ A couple of times a day

- Once a day
- A couple of times a week
- Less than once a week
- A couple of times a month
- Less than once a month
- Every other month
- I rarely engage with the group

Is the group moderated?

A moderator is a person who runs and manages the group, and is responsible for the smooth running of the group, including reviewing and approving posts and other content.

- Yes by professional service, either paid staff or volunteers
- Yes by peers in the group
- No
- I don't know

Does the group have a set of written rules about interacting with the group?

- Yes, you have to tick a box to agree to these before joining
- Yes but you don't have to actively agree to these before joining
- No
- I don't know

In your experience, what is the key benefit of being a member of an online support group? (Optional)

Now, thinking about this group, please answer the next set of questions.

I identify with others in this group

- ○ Strongly disagree
- ○ Somewhat disagree
- ○ Neither agree nor disagree
- ○ Somewhat agree
- ○ Strongly agree

I see myself as a member of this group

- ○ Strongly disagree
- ○ Somewhat disagree
- ○ Neither agree nor disagree

- Somewhat agree
- Strongly agree

I am glad to be a member of this group

- Strongly disagree
- Somewhat disagree
- Neither agree nor disagree
- Somewhat agree
- Strongly agree

I feel strong ties with this group

- Strongly disagree
- Somewhat disagree
- Neither agree nor disagree
- Somewhat agree
- Strongly agree

If someone criticized this group, it would feel like a personal insult.

- Strongly disagree
- Somewhat disagree
- Neither agree nor disagree

- ○ Somewhat agree
- ○ Strongly agree

I am very interested in what others think about this group

- ○ Strongly disagree
- ○ Somewhat disagree
- ○ Neither agree nor disagree
- ○ Somewhat agree
- ○ Strongly agree

When I talk about this group, I usually say 'we' rather than 'they'.

- ○ Strongly disagree
- ○ Somewhat disagree
- ○ Neither agree nor disagree
- ○ Somewhat agree
- ○ Strongly agree

This group's successes are my successes.

- ○ Strongly disagree
- ○ Somewhat disagree
- ○ Neither agree nor disagree

- ○ Somewhat agree
- ○ Strongly agree

If someone praised this group, it would feel like a personal compliment.

- ○ Strongly disagree
- ○ Dislike somewhat
- ○ Neither agree nor disagree
- ○ Somewhat agree
- ○ Strongly agree

If a story in the media criticized this group, I would feel embarrassed.

- ○ Strongly disagree
- ○ Somewhat disagree
- ○ Neither agree nor disagree
- ○ Somewhat agree
- ○ Strongly agree

Still thinking about this group and the support they provide you, how often is each of the following kinds of support available to you from this group?

	None of the time	A little of the time	Some of the time	Most of the time	All of the time	N/A
Someone you can count on to listen to you when you need to talk	○	○	○	○	○	○
Someone to give you information to help you understand a situation	○	○	○	○	○	○
Someone to give you good advice about a crisis	○	○	○	○	○	○
Someone to confide in or talk to about yourself or your problems	○	○	○	○	○	○
Someone whose advice you really want	○	○	○	○	○	○
Someone to share your most private worries and fears with	○	○	○	○	○	○
Someone to turn to for suggestions about how to deal with a personal problem	○	○	○	○	○	○
Someone who understands your problems	○	○	○	○	○	○
Someone to have a good time with	○	○	○	○	○	○
Someone to get together with for relaxation	○	○	○	○	○	○

	None of the time	A little of the time	Some of the time	Most of the time	All of the time	N/A
Someone to do something enjoyable with	○	○	○	○	○	○

Pathway B-do not use groups

Why are you not a member of online support groups?

Click all that apply

- ☐ I have enough support from family and friends
- ☐ I don't know any groups
- ☐ I'm not comfortable with being online in general
- ☐ I'm not comfortable using the platforms the groups are on
- ☐ I don't know how to find online support groups
- ☐ I am not interested
- ☐ I am concerned about privacy
- ☐ I was a member of a group but didn't find it useful
- ☐ I was a member of a group but had a bad experience and this put me off
- ☐ I have other priorities at the moment
- ☐ I don't have time
- ☐ [ ] Other

Would you be interested in joining an online support group for family caregivers?

- ○ Yes
- ○ No
- ○ Not sure

What factors would be important for you if you were joining an online support group?

Click all that apply.

For clarity, a moderator is a person who runs and manages the group, and is responsible for the smooth running of the group, including reviewing and approving posts and other content.

- ☐ The group is private
- ☐ The group is moderated by professional staff
- ☐ The group is moderated by volunteers
- ☐ The group is moderated by other members of the group
- ☐ The group is for family caregivers only
- ☐ I have the option to post without using my real name in the group
- ☐ There are many ways to engage with the group e.g. messaging, joining in video-chats, quizzes and competitions etc
- ☐ The group is specific to the condition of the person that I am caring for e.g. dementia specific, autism specific, etc

☐ [ ] Other

Perceived Stress

Thank you for all your responses so far.

The next 4 questions ask you about your feelings and thoughts during the LAST MONTH.
In each case, indicate by ticking in the appropriate space how often you felt or thought a certain way.
Although some of the questions are similar, there are differences between them and you should treat each one as a separate question.

In the past month, how often have you felt that you were unable to control the important things in your life?

- ○ Never
- ○ Almost never
- ○ Sometimes
- ○ Fairly Often
- ○ Very Often

In the past month, how often have you felt confident about your ability to handle personal problems?

- Never
- Almost Never
- Sometimes
- Fairly Often
- Very Often

In the past month, how often have you felt that things were going your way?

- Never
- Almost Never
- Sometimes
- Fairly Often
- Very Often

In the past month, how often have you felt difficulties were piling up so high that you could not overcome them?

- Never
- Almost Never
- Sometimes
- Fairly Often

○ Very Often

Caregiver_Background

In this second last section, we ask for information about your caregiving background.

This information helps us understand more about who is caregiving, why they are caregiving, the impact of caregiving on their lives and how this relates to online support group engagement.

How many people do you care for?

○ 1
○ 2
○ more than 2

What is your relationship to the person(s) you care for?
Click all that apply

☐ Parent/parent in law
☐ Son/Daughter or Son/Daughter-in-law

- [ ] Brother/sister or brother/sister-in-law
- [ ] Grandparent
- [ ] Aunt/Uncle
- [ ] Grandchild
- [ ] Friend/Neighbour
- [ ] Other

How many years have you been caring? If you care for more than one person, select the longest length of time.

Approximately how many hours a week do you provide care?

- ○ 1-5
- ○ 6-10
- ○ 11-15
- ○ 16-20
- ○ 21-30
- ○ 21-40
- ○ +40

As well as providing care, are you in employment/education outside the home?
Click all that apply.

- ☐ Yes, I work full time and provide care
- ☐ Yes, I work part time and provide care
- ☐ Yes, I work casual hours and provide care
- ☐ Yes, I study full time and provide care
- ☐ Yes, I study part time and provide care
- ☐ I volunteer and provide care
- ☐ I do not have time to work or study but would like to
- ☐ I do not wish to work or study
- ☐ I had to give up work or study because of my caring role
- ☐ None of the above

For what conditions/disabilities/illness does the person(s) need your care?
Click all that apply

- ☐ Physical disability
- ☐ Intellectual disability
- ☐ Autism Spectrum
- ☐ Mental Health Condition e.g. Anxiety, Depression
- ☐ Chronic Health Condition e.g. COPD
- ☐ Dementia/Alzheimers
- ☐ Aquired Brain Injury e.g. stroke
- ☐ Frailty due to aging
- ☐ Drug or alcohol dependency

☐ [ ] Other

Demographics

Finally, we need some demographic information.

This is used in the statistical reporting of the results of the survey, to contribute to an overall understanding of who is caregiving and how this relates to online support group engagement.

What age are you?

[ ]

What gender do you identify with?

- ○ Male
- ○ Female
- ○ Non-binary / third gender
- ○ Transgender
- ○ [ ] Other

○ Prefer not to say

What is your nationality?

[ ]

What country are you currently living in?

[ ]

Which option(s) below do you most identify with?

Click all that apply

- ☐ African
- ☐ American
- ☐ Arab
- ☐ Asian
- ☐ Black
- ☐ European
- ☐ Hispanic
- ☐ Middle Eastern
- ☐ Mixed racial or multi-ethnic background
- ☐ Traveller or Roma
- ☐ White

☐ None

☐ [ ] Other

☐ Prefer not to say

Is there anything you would like to include here that you feel is important and that we have not covered about Online Support Groups?

[ ]

A final reminder that if you would like to be contacted for follow-up from this survey, you can include your email address here.

[ ]

Powered by Qualtrics
